# Supplementary material for: Selective gating to vibrational modes through resonant X-ray scattering
Source: Nat Commun. 2017 Jan 20;8:14165. doi: 10.1038/ncomms14165 (PMC5263870; doi:10.1038/ncomms14165)
Supplement: Supplementary Information — Supplementary Figures, Supplementary Tables, Supplementary Notes and Supplementary References. [file ncomms14165-s1.pdf]

Supplementary Figures

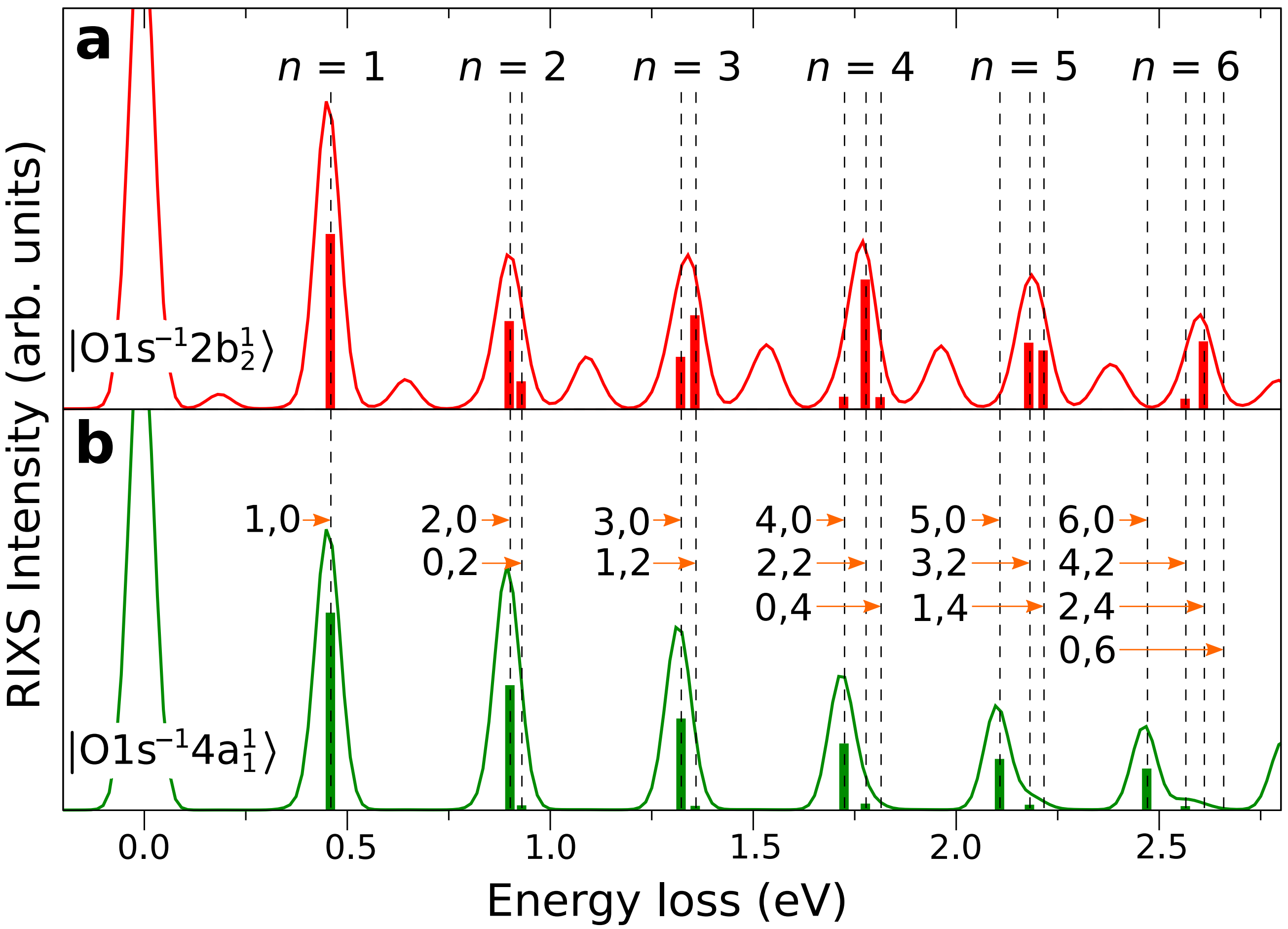

Supplementary Figure 1. Theoretical spectra of resonant inelastic X-ray scattering via $|\mathrm{O1s}^{-1}2\mathrm{b}_2^1\rangle$ and $|\mathrm{O1s}^{-1}4\mathrm{a}_1^1\rangle$ core-excited states. The spectra were calculated at detuning $\Omega = -0.025$ eV, and +0.05 eV from the top of the (**a**) $|\mathrm{O1s}^{-1}2\mathrm{b}_2^1\rangle$ and (**b**) $|\mathrm{O1s}^{-1}4\mathrm{a}_1^1\rangle$ absorption resonances, respectively. The dashed lines represents the ground state eigenvalues (see Supplementary Table 1). The colored bars below the spectra are the Franck-Condon amplitudes between the ground state wave functions and the core-excited wave packets $|\langle\psi_{\mathrm{n_s,n_a}}|\Phi_0\rangle|^2$.

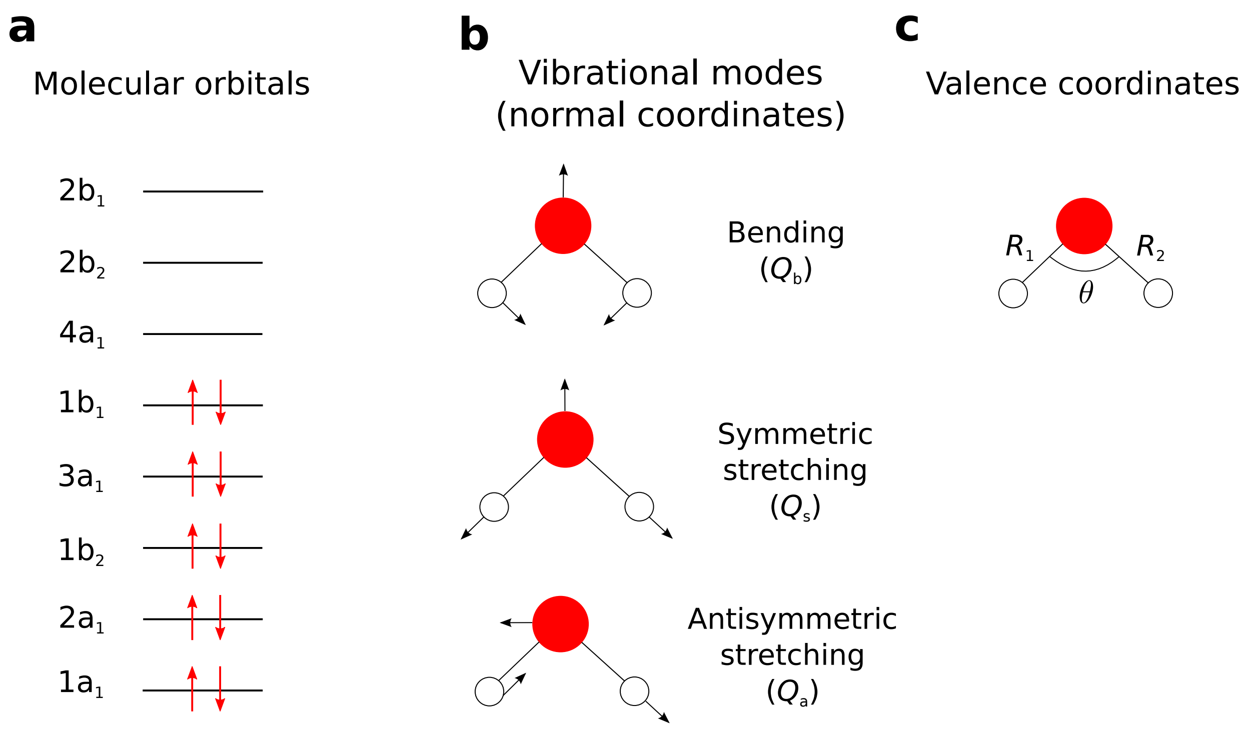

Supplementary Figure 2. Vibrational modes and molecules orbitals of water molecule. (**a**)The molecular orbitals are in energy order. (**b**) The water molecule has three vibrational normal modes: bending (b), symmetric (s) and antisymmetric (a) stretching. The experimental harmonic vibrational frequencies of the ground state are ω_b =1594.59 cm^{-1} =0.1977 eV, ω_s =3656.65 cm^{-1} =0.4534 eV, ω_a =3755.79 cm^{-1} =0.4657 eV [1]. (**c**) Valence coordinates representation used in the article.

Supplementary Tables

Supplementary Table 1. Theoretical vibrational states of gas-phase water. The vibrational levels of the 2D stretching potential energy surfaces ($\varepsilon_{\mathrm{n_s,n_a}}$) and 1D bending potential energy curve (ε_m) of the ground state H_2O (Fig. 1e) are compared with the experimental values taken from Ref. [2] (see Supplementary Notes 5 for computational details). The vibrational energies (in eV) are given relative to the zero-point energy, (0,0,0). The 2D stretching mode energies are represented using symmetric and antisymmetric modes representation ($n_\mathrm{s}, n_\mathrm{a}$). As it is discussed above, this approximation breaks down for large $n = n_\mathrm{s} + n_\mathrm{a}$.

n	(n_s,n_a)	Theory	Exp.	m	Theory	Exp.
1	(1,0)	0.4588	0.4531	1	0.1922	0.1976
	(0,1)	0.4680	0.4654			
2	(2,0)	0.9003	0.8923	2	0.3806	0.3907
	(1,1)	0.9037	0.8983			
	(0,2)	0.9295	0.9224			
3	(3,0)	1.3222	1.3133	3	0.5647	0.5786
	(2,1)	1.3229	1.3150			
	(1,2)	1.3581	1.3467			
	(0,3)	1.3763	1.3669			
4	(4,0)	1.7241	1.7133	4	0.7436	0.7605
	(3,1)	1.7242	1.7137			
	(2,2)	1.7769	1.7620			
	(1,3)	1.7869	1.7741			
	(0,4)	1.8141	1.8012			
5	(5,0)	2.1070	2.0937	5	0.9158	0.9351
	(4,1)	2.1070	2.0938			
	(3,2)	2.1807	2.1631			
	(2,3)	2.1841	2.1677			
	(1,4)	2.2153	2.1990			
	(0,5)	2.2408	-			

Supplementary Table 2. Theoretical geometry for the ground and core-excited states.

State	R (Å)	θ (°)
Ground	0.9588 [+0.0001]*	104.21 [+0.31]*
$\lvert O1s^{-1}2b_2^1\rangle$	1.0828	110.92
$\lvert O1s^{-1}2b_1^1\rangle$	0.9588	115.48

*Deviation from experiment [3].

Supplementary Notes 1: Details of the 2D+1D model

The strict solution of Schrödinger equation for the water molecule having three vibrational degrees of freedom (Supplementary Figure 2), can be obtained on a grid of three coordinates: R_1, R_2 and θ (angle $\angle$ HOH). Our software allows us to model the full 3D problem. However, the full 3D solution is rather expensive numerically, both for the electronic structure (3D *ab initio* potential energy surfaces (PES)) and wave packet calculations. It was pointed out a long time ago [4, 5] that the resonance coupling between the symmetric (s) and antisymmetric (a) stretching modes, named the Darling-Dennison coupling, is present in the water system. This is because their vibrational frequencies are close, $\omega_s = 3656.65$ cm^{-1} and $\omega_a = 3755.79$ cm^{-1} (Supplementary Figure 2), and due to the strong anharmonicity (Figs. 1c and 1e). There is also a Fermi resonance [4], which arises from the coupling between the bending and symmetric stretching modes. We ignore this rather weak coupling, because it is not as significant as the Darling-Dennison mode coupling in the case of X-ray scattering. Due to the strong stretching mode coupling and weak coupling to the bending mode, we developed a 2D+1D model, where we treat the bending mode (1D) separately from the two coupled stretching modes (2D) (Figs. 1b, 1c and 1e, and Supplementary Figure 2b). Following this model, we use an approximation with full 2D time-dependent consideration of the stretching modes, whereas the bending mode is treated within the 1D Franck-Condon (FC) formalism. The 2D+1D approximation follows naturally from specific mode coupling of water, described above.

The 2D stretching dynamics is obtained by the numerical solution of the time-dependent equation:

$$\imath \frac{\partial}{\partial t}\psi_j(t) = h_j \psi_j(t) \tag{1}$$

with $j =$c, f (c represents the core-excited and f the final state). We write the nuclear Hamiltonian in valence coordinates R_1, R_2 and θ_0 [6] (see Supplementary Figure 2c)

$$h_j = -\frac{1}{2\mu_1}\frac{\partial^2}{\partial R_1^2} - \frac{1}{2\mu_2}\frac{\partial^2}{\partial R_2^2} - \frac{\cos\theta_0}{m_{\mathrm{O}}}\frac{\partial^2}{\partial R_1 \partial R_2} + V_j(R_1, R_2, \theta_0) \tag{2}$$

where θ_0 is the equilibrium bond angle ($\theta_0 = 104.21°$) and $V_j(R_1, R_2, \theta_0)$ is the 2D PES. We introduce here the reduced masses

$$\mu_1 = \frac{m_1 m_{\mathrm{O}}}{m_1 + m_{\mathrm{O}}}, \tag{3}$$

$$\mu_2 = \frac{m_{\mathrm{O}} m_2}{m_{\mathrm{O}} + m_2}, \tag{4}$$

where $m_1 = m_2 = m_{\mathrm{H}}$. The bending mode contribution is treated by the solution of the time-independent 1D Schrödinger equation and the calculation of the FC amplitudes $\langle m_i | m_j \rangle$ between the bending vibrational levels m of the i and j electronic states, $i, j =$0, c, f (0 represents the ground state).

The resonant inelastic X-ray scattering (RIXS) cross section within the model is using eqs. (1) and (2). Since we focus here only on the spectral shape of the quasi-elastic RIXS (scattering to the ground electronic state, f = 0), the transition dipole moments $\mathrm{d_{fc}}$ and $\mathrm{d_{c0}}$ are equal and they are omitted in eqs. (1) and (2). All spectra are presented as function of the energy loss $\omega - \omega'$.

Supplementary Notes 2: Dependence of the RIXS vibrational profile on the polarization

The strength of a vibrational resonance in RIXS is defined by the FC amplitude, which does not depend on the polarization $\mathbf{e}$ in the Born-Oppenheimer (BO) approximation. The situation changes when the transition dipole moment $\mathbf{d}$ depends strongly on a molecular geometry due to the breakdown of the BO approximation. In this case, the factor $(\mathbf{e} \cdot \mathbf{d})$ should be included in the FC amplitude

$$\langle n' | n \rangle \to \langle n' | \mathbf{e} \cdot \mathbf{d} | n \rangle. \tag{5}$$

This brings an opportunity to affect the strength of a vibrational transition by varying the angle between polarizations of incoming and scattered X-ray photons due to dependence of the generalized FC amplitude (Supplementary Equation 5) on the polarization. However, the dependence of $\mathbf{d}$ on molecular geometry becomes significant only near the crossing of the potential energy surfaces of the excited electronic states. This effect was recently studied in articles devoted to RIXS in O_2 [7] and CO [8], as well as in the resonant Auger scattering from acetylene [9]. This non-BO effect is not the case for H_2O studied here, where the potentials of all considered core-excited states are nicely isolated. Due to this fact the polarization dependence of the vibrational profile is neglected in our simulations.

Supplementary Notes 3: Anharmonicity and complete breakdown of independent normal mode approximation

The anharmonicity of the PESs (Figs. 1c and 1e) is taken into account in our simulations explicitly, since the Schrödinger equation (Supplementary Equations 1 and 2) is solved strictly. We would like to pay attention to the anharmonicity of the 2D potential of the stretching motion. Let us note, that all the stretching PESs shown in Figs. 1c and 1e (including the ground state PES) deviates strongly from the elliptical shape, which corresponds to the harmonic PES. Moreover, the dissociative potential of the first core-excited state $|O1s^{-1}4a_1^1\rangle$ can not be described in principle by the harmonic approximation, where the nuclear motion is bound. This results in a strong coupling of the symmetric and antisymmetric vibrational modes, as it is described above. One should notice that the stretching modes (n_s, n_a) are grouped in the manifold of $n = n_s + n_a$ vibrational levels. In the independent normal-mode model, one can observe a monotonically varying energy spacing. In the H_2O molecule, however, the harmonic approximation is only valid for small $n = 0,1,2$, and fails for the higher groups starting from $n = 3$ (see Supplementary Table 1). In the nth group ($n \geq 3$), one can observe a degeneracy of the low lying vibrational energy levels caused by the anharmonic mode coupling

$$\varepsilon_{n,0} \approx \varepsilon_{n-1,1}. \quad (6)$$

Another manifestation of strong anharmonicity of 2D potentials is the deviation of the shape of strict vibrational wave functions from the wave functions in harmonic approximation (see Fig. 2c-h). Thus, the strong anharmonicity of the 2D PESs of the ground and core-excited states motivates us to describe the stretching dynamics explicitly without using the independent mode approximation. To the best our knowledge, in the contrast to the present article, all previous RIXS studies of polyatomic molecules and solids were performed in the framework of the independent mode approximation. The studied RIXS spectrum of the water molecule is a nice showcase where the independent mode approximation is fully broken.

Supplementary Notes 4: RIXS via $|O1s^{-1}2b_1^1\rangle$ core-excited state: role of the Thomson scattering

The Rayleigh or zero energy loss (ZEL) peak ($\omega = \omega'$) is strongly affected by the Thomson scattering with the amplitude [10–12]

$$F_T = Z(\mathbf{e}' \cdot \mathbf{e})\delta_{\nu_f,0} \quad (7)$$

where Z is the number of electrons in molecule, $\mathbf{e}$ and $\mathbf{e}'$ are the polarization vectors of incoming and scattered X-ray photons. The Thomson scattering is ignored in our simulations because our main attention is paid to the scattering to the excited vibrational level of the final electronic state. In spite of this, the Thomson scattering can affect the intensity of resonances lying close to the ZEL peak. The role of the Thomson scattering is seen clearly from comparison of our simulations with the experimental RIXS spectra. Figure 2a of the article shows that the theory underestimates the intensity of the ZEL peak for all core-excited states, due to dominant contribution of the Thomson scattering to the intensity of this resonance. In the case of the $|O1s^{-1}2b_1^1\rangle$ RIXS spectrum, the tail of the experimental ZEL peak contributes much to the intensity of the next closely-lying bending resonance $m = 1$. The stretching frequencies are twice bigger as compared to the bending one (see Supplementary Table 1), and due to fact the contribution of the Thomson scattering to the stretching quanta $n = 1$ is much smaller. This explains why our simulations underestimate the intensity of the peak $m = 1$ at the $|O1s^{-1}2b_1^1\rangle$ RIXS spectra.

One should notice that the experimental $|O1s^{-1}2b_1^1\rangle$ RIXS spectrum is more noisy than the $|O1s^{-1}2b_2^1\rangle$ and $|O1s^{-1}4a_1^1\rangle$ RIXS spectra. The main reason for the noisy spectrum is the rather large detuning from the narrow $|O1s^{-1}2b_1^1\rangle$ resonance $\Omega = 0.2$ eV. This makes the intensity of the corresponding RIXS band smaller and hence more noisy.

Supplementary Notes 5: Theoretical vibrational frequencies

The stretching vibrational states of the ground state of H_2O (Supplementary Table 1) were obtained by numerical solution of the time-independent Schrödinger equation

$$h\psi = \varepsilon\psi, \quad (8)$$

with the 2D Hamiltonian given by Supplementary Equation (2) and the *ab initio* PES. The same approach was used to compute bending vibrational frequencies and wave functions for all electronic states using 1D nuclear Hamiltonian for the bending coordinate and the *ab initio* potentials.

Supplementary Notes 6: Details of calculation of the potential energy surfaces

Q_s The equilibrium geometry and normal vibrational modes of ground state water were obtained by a RASPT2 calculation considering an active space of eight electrons distributed in nine orbitals, with the ANO-RCC basis set (see Methods). Calculated potential energy curves (1D) of the bending vibrational mode and stretching potential energy surfaces (2D) as a function of bond lengths $R_1 = R_{OH_1}$ and $R_2 = R_{OH_2}$ for the ground and core-excited states are presented in Fig. 1. Vibrational frequencies of the ground electronic state of H_2O of stretching and bending modes are collected in Supplementary Table 1, where our calculations are compared against the experimental data. Our simulations of the equilibrium geometry of the ground state $R_{OH} = 0.9588$ Å and $\angle(\text{HOH}) = 104.21°$ are in good agreement with the well established experimental values ($R_{OH} = 0.9587$ Å and $\angle(\text{HOH}) = 103.9°$ [3]). The equilibrium values for all electronic states used in our paper are summarized in Supplementary Table 2, except the dissociative state $|\text{O1s}^{-1}4\text{a}_1^1\rangle$ which apparently has no equilibrium geometry.

Supplementary References

1. Tennyson, J. *et al.* IUPAC critical evaluation of the rotational–vibrational spectra of water vapor, Part III: Energy levels and transition wavenumbers for $H_2^{16}O$. *J. Quant. Spectrosc. Radiat. Transfer* **117** 29–58 (2013).

2. Császár, A. G. et al. First-principles prediction and partial characterization of the vibrational states of water up to dissociation. *J. Quant. Spectrosc. Radiat. Transf.* **111**, 1043–1064 (2010).

3. Cook, R.L., DeLucia, F.C. & Helminger, P. Molecular force field and structure of water: Recent microwave results. *J. Mol. Spectrosc.* **53**, 62–76 (1974).

4. Herzberg, G. & Spinks, J. *Molecular Spectra and Molecular Structure: Part I. Spectra of Diatomic molecules* (Van Nostrand, 1989).

5. Darling, B. T. & Dennison, D. M. The Water Vapor Molecule. *Phys. Rev.* **57**, 128–139 (1940).

6. Jensen, P. Hamiltonians for the internal dynamics of triatomic molecules. *J. Chem. Soc., Faraday Trans. 2* **84**, 1315–1339 (1988).

7. Hennies, F. *et al.* Resonant Inelastic Scattering Spectra of Free Molecules with Vibrational Resolution. *Phys. Rev. Lett.* **104**, 193002 (2010).

8. Couto, R. C. *et al.* Anomalously strong two-electron one-photon X-ray decay transitions in CO caused by avoided crossing. *Sci. Rep.* **6**, 20947 (2016)

9. Miron, C. *et al.* Vibrational Scattering Anisotropy Generated by Multichannel Quantum Interference. *Phys. Rev. Lett.* **105**, 093002 (2010).

10. Gel'mukhanov, F. & Ågren, H. Theory of resonant elastic X-ray scattering by free molecules. *Phys. Rev. A* **56**, 2676–2684 (1997).

11. Gel'mukhanov, F. & Ågren, H. Resonant X-ray Raman scattering. *Phys. Rep.* **312**, 87–330 (1999).

12. Pietzsch, A. *et al.* Spatial Quantum Beats in Vibrational Resonant Inelastic Soft X-ray Scattering at Dissociative States in Oxygen. *Phys. Rev. Lett.* **106**, 153004 (2011).
